# Morphological Variations of the Pterygomaxillary Suture According to Skeletal Patterns

**DOI:** 10.3390/diagnostics15192467

**Published:** 2025-09-26

**Authors:** Tuğçe Akın, Hacer Eberliköse, Berin Tuğtağ Demir, Burak Bilecenoğlu, Hakan Alpay Karasu

**Affiliations:** 1 Department of Clinical Anatomy, Institute of Health Sciences, Istanbul Medipol University, Istanbul 34810, Turkey; 2 Department of Anatomy, Faculty of Medicine, Ankara Medipol University, Ankara 06570, Turkey; berin.tugtag@ankaramedipol.edu.tr; 3 Department of Oral and Maxillofacial Surgery, Faculty of Dentistry, Ankara Medipol University, Ankara 06570, Turkey; hacer.eberlikose@ankaramedipol.edu.tr (H.E.); hakan.karasu@ankaramedipol.edu.tr (H.A.K.); 4 Department of Anatomy, Institute of Health Sciences, Istanbul Medipol University, Istanbul 34810, Turkey

**Keywords:** cone-beam computed tomography, orthognathic surgery, dentofacial abnormalities

## Abstract

**Background:** The posterosuperior maxillary region poses a challenge in orthognathic surgery due to its complex three-dimensional anatomy. The pterygomaxillary suture (PMS) is a key landmark for various procedures. Understanding its anatomical relationships is essential to improving surgical precision. **Methods:** A retrospective analysis of CBCT images from 120 patients aged 18–70 years at Ankara Medipol University was conducted. Patients were categorized into skeletal Classes I, II, and III according to the ANB angle. Linear and angular measurements of the PMS and adjacent structures were performed. The statistical analysis included the Shapiro–Wilk, Independent *t*-test, Mann–Whitney U test, and regression analysis (*p* < 0.05). **Results:** There were clear differences between the skeletal groups. Class II and III patients had a lesser lateral PMS–baseline intersection distance (IV–VI) and Class II had a lesser medial PMS–baseline perpendicular distance (VV′) compared to Class I (*p* < 0.05). Additionally, the angle V–IV–VI was significantly narrower in Class II and III groups, indicating altered PMS orientation in these skeletal patterns. **Conclusions:** PMS morphology, including thickness, width, and angulation, is influenced by skeletal pattern. A preoperative CBCT assessment and individualized surgical planning are essential to ensure the safety and accuracy of Le Fort I osteotomies, especially in Class II and III patients.

## 1. Introduction

The posterosuperior maxillary region is of particular significance in orthognathic surgery due to its deep location, poor visualization, and proximity to critical anatomical structures. This region carries a high risk of bleeding because of its dense vascularization, which further complicates surgical procedures [1]. The pterygomaxillary suture (PMS) is an important landmark in this region for maxillary nerve blocks, tumor resections of the posterior maxilla and skull base, and Le Fort I osteotomy [2]. Anatomically, the PMS forms a critical junction between the craniofacial skeleton and neighboring fossae by joining the maxilla to the lateral pterygoid plate of the sphenoid bone [3].

Previous studies have investigated PMS morphology using Computed tomography (CT) and cone-beam computed tomography (CBCT), focusing on parameters such as suture height, width, thickness, and their relationship to surrounding structures [4,5,6]. Some of these reports have emphasized ethnic variation, while others have examined its implications for osteotomy techniques [7,8]. However, these studies did not systematically evaluate PMS morphology according to skeletal classification. This constitutes an important gap, because skeletal class (I, II, III) is a routine diagnostic parameter in orthodontics and orthognathic surgery and is directly relevant for preoperative planning. Understanding how PMS morphology varies with skeletal pattern is therefore more clinically relevant than ethnicity-based differences, as it can directly guide surgical access, osteotome angulation, and complication risk.

In Class II patients, reduced PMS dimensions may hinder osteotomy access and increase the risk of unfavorable fracture lines, whereas in Class III patients, broader posterior maxillary morphology may necessitate deeper osteotome insertion and adjusted angulation [5]. These differences highlight the importance of a skeletal class–specific evaluation of PMS morphology. Accordingly, the present study aims to provide a comprehensive CBCT-based morphometric analysis of PMS across skeletal Classes I, II, and III. The null hypothesis tested was that skeletal pattern exerts no significant effect on PMS morphology.

## 2. Materials and Methods

A total of 120 patients, aged 18–70 years and presenting with different skeletal patterns (Class I, II, III according to the ANB angle), were included in this retrospective study. The study’s participants were exclusively drawn from the Turkish population. All eligible patients who met the inclusion and exclusion criteria were included consecutively during the study period, without additional stratification, to minimize selection bias. Ethical approval for this retrospective study was obtained from the Istanbul Medipol University Ethics Committee (Protocol No. 178) in early 2025, prior to the submission of the manuscript. CBCT scans from patients who visited the Oral and Maxillofacial Surgery Department at Ankara Medipol University Faculty of Dentistry between 2023 and 2024 were retrospectively analyzed to assess linear and angular measurements of the pterygo-maxillary suture (PMS) and adjacent anatomical structures.

All scans were obtained using the same device (Castellini, X Radius TrioPlus, Imola (BO), Italy) under standardized settings: The parameters for the image acquisition included 90 kVp, 8 mA, a voxel size of 0.2 mm^3^, a slice thickness of 1 mm, and a wide field of view (18 × 16 cm). In sagittal images, the Frankfort horizontal plane was positioned parallel to the floor, ensuring alignment with the software’s inherent horizontal plane. Two-dimensional (2D) images were generated from horizontal sections aligned with the zygomaticoalveolar line, which closely corresponds to the LF1 reference line. Standardization was imperative to ensure precise and consistent measurements across all scans.

Patients with a documented history of craniofacial syndrome or congenital anomalies, tumors, prior orthognathic surgery and cleft palate, cranial trauma, age less than 18 years, age over 70 years, and CBCT images with artifacts in the area to be measured were excluded from the study. Epidemiological data, encompassing age, sex, and the presence or absence of third molars, were extracted from patients’ medical records.

The Steiner analysis was employed to ascertain the A-N-B angle. Angles greater than 4° were classified as skeletal Class II, while those below 0° were categorized as skeletal Class III.

Axial CBCT images were analyzed to assess the lengths and angles of the maxilla and pterygoid plates, as well as their positional relationship and pterygomaxillary suture just beneath the level of the inferior nasal concha and approximately 3 to 5 mm above the nasal floor.

**Figure 1:**
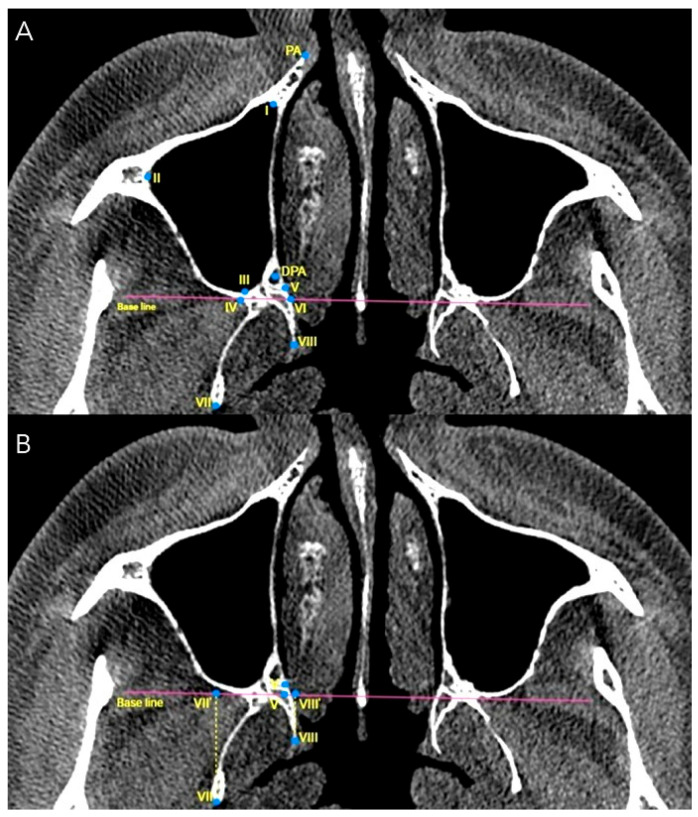
The anatomical measurement points around the pterygomaxillary suture in axial view (**A**,**B**) (I: Most anterior point on the internal surface of the maxillary sinus; II: Most lateral point on the internal surface of the maxillary sinus; III: Most posterior point on the internal surface of the maxillary sinus; IV: Deepest concavity between posterior maxilla and lateral pterygoid plate (lateral PMS point); Baseline: Line connecting the bilateral ‘IV’ points; V: Medial point of the pterygomaxillary suture; V′: Perpendicular projection of point ‘V’ onto the baseline; VI: Intersection of the baseline and medial pterygoid plate; VII: Posterior point of the lateral pterygoid plate; VII′: Perpendicular projection of point ‘VII’ onto the baseline; VIII: Posterior point of the medial pterygoid plate; VIII′: Perpendicular projection of point ‘VIII’ onto the baseline; PA: Margin of the piriform aperture; DPA: Location of the descending palatine artery).

**Figure 2:**
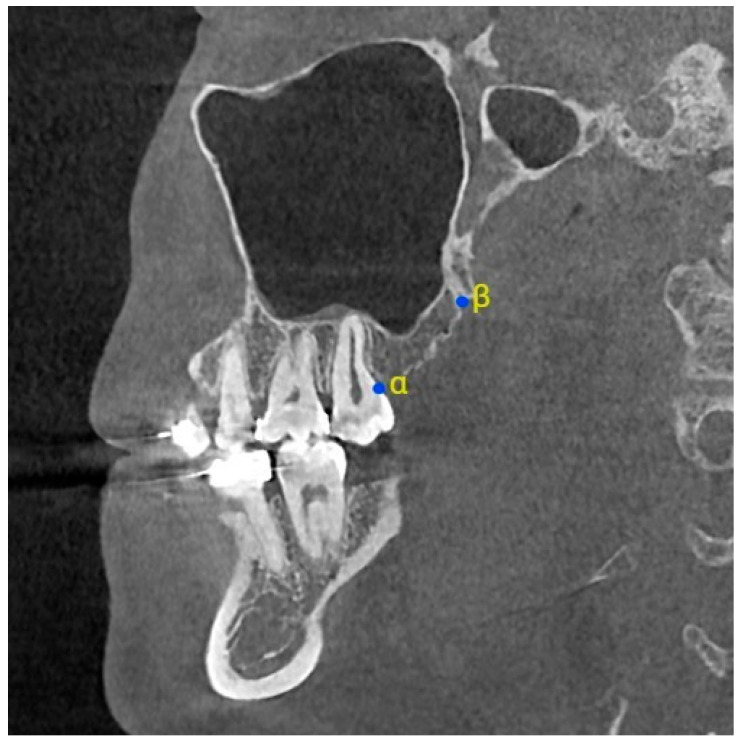
Measurement points in sagittal view. (α: Intersection of alveolar crest and distal surface of terminal molar; β: Lower margin of PMS).

The measurement points and reference lines employed for image analysis are presented in Table 1, Figure 1 and Figure 2. These were selected based on their relevance to surgical planning and clinical relevance in the posterior maxillary region, particularly in procedures such as posterior maxillary segmental osteotomies, pterygoid disjunction, and sinus floor elevation. It is imperative to recognize the significance of specific anatomical landmarks during surgical procedures. Points such as the deepest concavity between the maxilla and lateral pterygoid plate (Point IV) and the intersection of the pterygoid plates (Points VII and VIII) are crucial for avoiding vascular and neurovascular structures. Angles between the posterior wall of the maxillary sinus and the lateral pterygoid plate are crucial for spatial orientation, which is essential for minimizing surgical complications. The utilization of baseline and perpendicular projections (e.g., V′, VII′, VIII′) has been instrumental in standardizing measurements and enhancing reproducibility in clinical imaging. Furthermore, VIII–VIII′ distance reflects the posterior dimension of the medial pterygoid plate in relation to the baseline. This parameter is critical because reduced values indicate a compact posterior maxillary space, which has been associated with an increased risk of unfavorable fracture lines during Le Fort I osteotomy. PA–DPA distance represents the spatial relationship between the piriform aperture and the descending palatine artery. This measurement is essential for evaluating the proximity of osteotomy lines to major vascular structures and thus provides valuable information for minimizing the risk of intraoperative bleeding.

Overall, all selected reference points (e.g., IV, V, VII, VIII, PA, DPA) correspond to reproducible skeletal landmarks that are easily identifiable on CBCT scans and are commonly encountered during pterygoid disjunction or posterior maxillary osteotomies. Their inclusion allows for a standardized and clinically applicable morphometric analysis that can directly inform surgical decision-making.

Maxillary sinus size and volume were also assessed, as their proximity to the PMS may influence osteotomy angulation and surgical safety.

### Data Analysis

The statistical analysis was conducted using IBM SPSS Statistics version 21.0 (IBM Corp., Armonk, NY, USA). The Shapiro–Wilk test was applied to assess the normality of the data distribution. Subsequently, a one-way ANOVA test was employed to compare the measurements obtained from patients classified into the Class I, Class II, and Class III categories. For post-hoc analyses, the Bonferroni correction was implemented when homogeneity of variances was confirmed, whereas Tamhane’s T2 test was employed in cases of unequal variances. A series of comparisons were addressed by implementing statistical corrections where applicable. In the pursuit of identifying the factors that give rise to anatomical variations, the cut-off values were determined through the implementation of receiver operating curves, subsequently followed by logistic regression analysis. A *p*-value less than 0.05 was considered statistically significant. The interobserver and intraobserver variability were defined, and the intraclass correlation coefficient rates were determined to be 0.88 and 0.91, respectively.

For intraobserver reliability, measurements were repeated by the same observer with a two-week interval. For interobserver reliability, measurements were performed by two different observers on the same day at the same time.

## 3. Limitations of the Study

Relying exclusively on CBCT presents a limitation, as it does not capture variations in soft tissue. It is imperative that future studies integrate CBCT with MRI or histologic analysis to facilitate a comprehensive anatomical assessment. Moreover, these studies must investigate the impact of these variations on postoperative healing and long-term outcomes. The use of CBCT data has been demonstrated to enhance the efficacy of 3D-printed osteotomes and surgical simulation, thereby promoting precision and safety in surgical procedures.

Although we acknowledge the limitations of relying on ANB alone—such as its sensitivity to cranial base length and rotation—it was selected to ensure standardization and comparability with previous studies. Additional cephalometric indicators (e.g., Wits appraisal, SNA, SNB) were not included in order to avoid confounding factors and to keep the focus of the study strictly on PMS morphometry, rather than on overall craniofacial relationship.

While the present study identifies significant morphometric differences of the pterygo-maxillary suture among skeletal patterns, the correlation of these findings with intraoperative or postoperative outcomes, such as occurrence of fracture or surgical complications, was not the objective of this study. This limitation is explicitly articulated, and we underscore the necessity for additional clinical studies to establish a direct correlation between anatomical variations and surgical risk and outcomes.

## 4. Results

The demographic characteristics and skeletal pattern distribution of the 120 patients are presented in Table 2. Descriptive statistics of the pterygomaxillary measurements for Class I, II, and III skeletal patterns are summarized in Table 3.

Overall comparisons revealed significant differences among the three skeletal classes in several measurements, including V–V′, VIII–VIII′, PA–DPA, α–β, ∠V–IV–VI, ∠II–I–III, ∠I–II–III, ∠I–III–II, I–III, and ∠VI–IV–VII (*p* < 0.05).

Post-hoc analyses indicated that Class II patients had significantly shorter V–V′ (*p* = 0.012) and VIII–VIII′ (*p* < 0.001) distances compared to Class I and III, suggesting a more compact posterior maxillary region. In contrast, Class II patients showed significantly larger PA–DPA (*p* = 0.030) and Class III showed larger α–β (*p* = 0.032) distances, indicating a more pronounced separation in the pterygomaxillary region. The ∠V–IV–VI angle was narrower in Class II compared to both Class I and Class III (*p* = 0.013). Conversely, angles ∠II–I–III (*p* < 0.001), ∠I–II–III (*p* = 0.003), and ∠I–III–II (*p* = 0.004) were narrower in Class II patients. Additional differences were also observed for the I–III distance (*p* = 0.041) and the ∠VI–IV–VII angle (*p* = 0.036), which followed patterns consistent with skeletal classification.

### Analysis of Risk Factors for the Occurrence of Skeletal Pattern Using Multinomial Logistic Regression

Multinomial logistic regression analysis was employed to identify cut-off values that were predictive of skeletal pattern occurrence. Only clinically relevant measurements showing significant differences between skeletal classes were included in the multinomial logistic regression. The cut-off values for V–V′ and VIII–VIII′ were determined from the dataset, and their corresponding odds ratios and confidence intervals indicate the likelihood of a skeletal pattern being Class II or III.

The findings indicate that a V–V′ distance less than 3.44 mm (OR 2.74, 95% CI 0.048–0.836, *p* = 0.015) and an VIII–VIII′ distance less than 12.9 mm (OR 2.79, 95% CI 1.043–1.958, *p* < 0.001) are associated with Class II skeletal pattern. For Class III patients, the following metrics were found to be significant predictors: PA–DPA (95% CI 1.055–1.614, *p* = 0.014) and α–β distances (95% CI 0.788–0.970, *p* = 0.012). These findings underscore the distinctive anatomical characteristics inherent to each skeletal class (see Table 4 for a detailed breakdown).

## 5. Discussion

This study rejects the null hypothesis, demonstrating that bone class significantly affects both linear and angular PMS measures. Patients classified as Class II and Class III exhibited unique morphological landmarks that directly influence surgical planning. Class II patients demonstrated reduced V–V′ and VIII–VIII′ lengths, as well as narrower V–IV–VI angles, signifying a condensed posterior maxillary area. These characteristics limit osteotome angulation and elevate the chance of adverse fracture lines, elucidating why Class II cases present greater therapeutic challenges. Conversely, Class III patients exhibited increased PA–DPA and α–β distances, indicating a more pronounced posterior morphology. This requires deeper insertion of the osteotome and meticulous angulation to prevent vascular damage, especially to the descending palatine artery. The findings emphasize that surgical planning for Le Fort I osteotomy should be tailored: conservative and regulated disjunction for Class II, in contrast to more profound and angle-modified disjunction for Class III.

Our findings align with those of Obata et al. [6], who indicated that diminished PMS thickness and adverse angulations elevate fracture risk, especially pertinent to Class II patients. Dadwal et al. [9], similarly underscored the necessity for technique modification in instances of restricted PMS thickness, whereas Lee et al. [10] demonstrated that increased PMS dimensions exacerbate surgical challenges in cleft patients—mirroring the surgical complexity noted in Class III cases. Odabaşı et al. [11] and Ueki et al. [12] similarly recorded changes in DPA and pterygoid plate morphology based on skeletal type, corroborating our observation of a greater PA–DPA distance in Class III patients. Conversely, Ayhan et al. [13] found no definitive association between PMS morphology and fracture pattern, indicating that various anatomical elements interact to influence surgical results. This study demonstrates that skeletal categorization is a critical factor that must be considered in preoperative planning.

Incorporating sinus volume throughout the research yielded further insight, as sinus shape is linked to PMS architecture and surgical accessibility. Alterations in sinus dimensions can modify osteotomy angulation, influence PMS orientation, and elevate situations such as sinus wall fracture or postoperative sinusitis. Consequently, sinus parameters were integrated to enhance the evaluation of anatomical risk factors during Le Fort I osteotomy.

In addition to skeletal class, gender and age also affect PMS morphology. Our observations of increased PMS dimensions in males align with the findings of Icen et al. [14], although age-related volumetric alterations noted by Icen and Orhan [15] highlight the necessity for personalized three-dimensional assessment. Collectively, these findings indicate that PMS morphology is heterogeneous, influenced by skeletal, gender, and age-related factors.

These discoveries have direct clinical consequences. In Class II patients, the use of thinner osteotomes and reduced disjunction forces may help mitigate fracture risk. The potential role of piezosurgery in reducing such risks remains to be clarified in future studies. In Class III patients, it is crucial to implement deeper osteotome placement and angle modification to prevent vascular injury. CBCT-derived morphometric data ought to be systematically incorporated into preoperative planning, and its integration into virtual surgical planning software can enhance osteotomy trajectories. Employing specialized devices, such as angled or ultrasonic osteotomes, could be beneficial in anatomically intricate instances, although this was not directly tested in the present study and warrants further investigation.

This study offers new, class-specific insights into PMS morphology, indicating that skeletal pattern is a therapeutically relevant determinant. These findings underscore the necessity of transitioning from a standardized surgical methodology to customized osteotomy techniques, thereby improving both the safety and accuracy of orthognathic surgery.

## 6. Conclusions

The present study provides surgical recommendations to enhance the safety and precision of Le Fort I osteotomies, focusing on Class II and III skeletal types. It has been demonstrated that variations in PMS thickness, width, and angulation, particularly in Class III patients, have been shown to increase the risk of fracture. Consequently, preoperative planning should adjust osteotomy angles based on CBCT measurements. The utilization of thinner or angled osteotomes, ultrasonic piezo surgery, and personalized cutting techniques has been demonstrated to reduce the incidence of complications. Furthermore, CBCT-based morphometric data can be integrated into virtual planning software for real-time osteotomy simulation in complex cases.

## Figures and Tables

**Table 1:** Anatomical Measurement Reference Table.

Point/Parameter	Plane	Description
I	Horizontal	Most anterior point on the internal surface of the maxillary sinus
II	Horizontal	Most lateral point on the internal surface of the maxillary sinus
III	Horizontal	Most posterior point on the internal surface of the maxillary sinus
IV	Horizontal	Deepest concavity between posterior maxilla and lateral pterygoid plate (lateral PMS point)
Baseline	Horizontal	Line connecting the bilateral ‘IV’ points
V	Horizontal	Medial point of the pterygomaxillary suture
V′	Horizontal	Perpendicular projection of point ‘V’ onto the baseline
VI	Horizontal	Intersection of the baseline and medial pterygoid plate
VII	Horizontal	Posterior point of the lateral pterygoid plate
VII′	Horizontal	Perpendicular projection of point ‘VII’ onto the baseline
VIII	Horizontal	Posterior point of the medial pterygoid plate
VIII′	Horizontal	Perpendicular projection of point ‘VIII’ onto the baseline
PA	Horizontal	Margin of the piriform aperture
DPA	Horizontal	Location of the descending palatine artery
α (Alpha)	Sagittal	Intersection of alveolar crest and distal surface of terminal molar
β (Beta)	Sagittal	Lower margin of PMS (corresponding to point IV)
I–II	Horizontal	Distance between I and II
II–III	Horizontal	Distance between II and III
III–I	Horizontal	Distance between III and I
IV–V	Horizontal	Distance between IV and V
IV–VI	Horizontal	Distance between IV and VI
IV–VII	Horizontal	Distance between IV and VII
VI–VIII	Horizontal	Distance between VI and VIII
VII–VIII	Horizontal	Distance between VII and VIII
IV–V′	Horizontal	Distance between IV and V′
V–V′	Horizontal	Distance between V and V′
IV–VII′	Horizontal	Distance between IV and VII′
VII–VII′	Horizontal	Distance between VII and VII′
VI–VIII′	Horizontal	Distance between VI and VIII′
VIII–VIII′	Horizontal	Distance between VIII and VIII′
PA–Baseline	Horizontal	Distance from PA to baseline
DPA–Baseline	Horizontal	Distance from DPA to baseline
PA–DPA	Horizontal	Distance between PA and DPA
α–β	Sagittal	Distance between α and β
∠II–I–III	Horizontal	Angle between points II–I–III
∠I–II–III	Horizontal	Angle between points I–II–III
∠I–III–II	Horizontal	Angle between points I–III–II
∠IV–VI–VIII	Horizontal	Angle between points IV–VI–VIII
∠V–IV–VI	Horizontal	Angle between points V–IV–VI
∠VI–IV–VII	Horizontal	Angle between VI–IV–VII
∠Lateralmedial pterygoid plates	Horizontal	Angle between pterygoid plates
∠Posterior surface of maxillary sinuslateral pterygoid plate	Horizontal	The deepest angle between the posterior surface of the maxillary sinus and the lateral pterygoid plate
Maxillary sinus area	Horizontal	Computed area of the maxillary sinus
Maxillary sinus volume	Horizontal	Computed volume of the maxillary sinus
Maxillary sinus width	Horizontal	The longest distance perpendicular from the most prominent point of the medial wall to the most prominent point of the lateral wall

**Table 2:** Patient demographics.

Age (years)	35.5 ± 20.5
Gender, *n* (%)	
Male	46 (38.3%)
Female	74 (61.7%)
Skeletal pattern	
Class I, *n* (%)	39 (32.5%)
Class II, *n* (%)	31 (25.8%)
Class III, *n* (%)	50 (41.7%)

**Table 3:** Clinical characteristics and anatomical measurements of the patients overall and by study group.

	Class I	Class II	Class III	*p*-Value
I–II (mm)	23.85 ± 4.34 (16.50–36.40)	25.68 ± 4.63 (18.90–34.45)	25.42 ± 6.60 (12.70–35.55)	0.065
II–III (mm)	27.93 ± 2.96 (20.50–34)	29.45 ± 3.30 (25.20–37.30)	27.15 ± 4.18 (19.40–34.45)	0.254
III–I (mm)	32.06 ± 3.35 (24.95–42.15)	33.32 ± 2.04 (29.90–35.40)	31.12 ± 3.99 (19.85–42.15)	0.041 ^(II–III)^ *
∠II–I–III (◦)	132.55 ± 21.88 (46.05–132.55)	62.13 ± 12.56 (85.30–122.20)	80.59 ± 13.53 (75.80–129.80)	0.000 ^(II–I, III)^ *
∠I–II–III (◦)	90.20 ± 12.97	84.22.15 ± 11.97	98.43 ± 15.33	0.003 ^(II–I)^ *
∠II–III–I (◦)	106.57 ± 10.03 (108.40–152.95)	63.56 ± 15.60 (94.65–149.25)	88.85 ± 11.07 (107.20–151.70)	0.004 ^(II–I)^ *
Maxillary sinus volume (10^3^ mm^3^)	14.89 ± 4.57 (7.58–30.77)	15.20 ± 8.28 (8.03–36.73)	16.72 ± 6.13 (6.03–27.79)	0.255
Maxillary sinus area (10 mm^2^)	48.10 ± 10.56 (29.76–87.95)	53.75 ± 13.56 (43.73–87.87)	49.24 ± 15.67 (23.42–78.87)	0.056
IV–V (mm)	7.52 ± 1.21 (5–9.94)	8.98 ± 2.24 (4.31–17.16)	7.77 ± 1.64 (4.58–10.80)	0.629
IV–VI (mm)	8.19 ± 1.27 (5.30–10.00)	6.01 ± 2.33 (5.03–12.37)	6.02 ± 1.73 (4.61–10.86)	0.025 ^(I–II, III)^ *
IV–VII (mm)	12.33 ± 2.81 (6.76–18.60)	12.72 ± 3.50 (7.33–13.60)	10.82 ± 2.91 (8.03–19.05)	0.072
VI–VIII (mm)	7.73 ± 2.26 (5.11–15.80)	9.35 ± 2.51 (5.50–13.20)	7.23 ± 1.91 (3.83–10.95)	0.689
VII–VIII (mm)	11.16 ± 2.29 (6.10–15.60)	10.49 ± 1.93 (8.11–13.55)	11.50 ± 3.17 (7.73–17.70)	0.682
IV–V′ (mm)	6.87 ± 1.18 (4.33–9.76)	6.48 ± 1.83 (3.37–9.02)	9.31 ± 1.20 (4.21–23.67)	0.431
IV–VII′ (mm)	4.06 ± 2.73 (0.63–14.95)	3.49 ± 2.37 (0.60–8.96)	4.64 ± 1.65 (1.44–28.80)	0.001
VI–VIII′ (mm)	0.93 ± 0.64 (0.00–2.24)	0.86 ± 0.45 (0.23–2.35)	1.58 ± 0.98 (0.05–9.90)	0.432
V–V′ (mm)	2.71 ± 0.68 (1.34–4.52)	1.77 ± 1.02(0.64–3.74)	2.60 ± 0.98 (0.47–8.90)	0.012 ^(II–I)^ *
VII–VII′ (mm)	11.62 ± 2.45 (6.53–17.20)	8.23 ± 3.45 (7.16–17.00)	10.63 ± 2.39 (7.48–18.00)	0.000 ^(II–I, III)^ *
VIII–VIII′ (mm)	7.88 ± 2.54 (3.47–16.76)	7.41 ± 2.75 (5.39–7.30)	7.05± 1.97 (3.71–11.10)	0.000 ^(II–I, III)^ *
∠IV–VI–VIII (◦)	87.19 ± 9.09 (68.40–115.25)	90.25 ± 6.85 (77.70–101.95)	87.04 ± 13.09 (70.50–114.90)	0.431
∠V–IV–VI (◦)	20.73 ± 5.16 (11.80–30.15)	15.04 ± 19.62 (4.60–76.00)	15.85 ± 4.92 (3.25–22.50)	0.013 ^(I–II, III)^ *
∠VI–IV–VII (◦)	105.13 ± 11.35 (63.40–119.40)	105.25 ± 11.52 (88.85–133.80)	109.81 ± 17.50 (58.65–125.85)	0.036 ^(III–I, II)^ *
∠Lateral–medial pterygoid plates (◦)	121.51 ± 12.10 (101.05–149.30)	119.75 ± 18.86 (79.75–147.15)	121.05 ± 12.75 (96.75–143.95)	0.996
∠Posterior surface of maxillary sinus–lateral pterygoid plate (◦)	98.77 ± 22.09 (55.85–148.00)	100.62 ± 23.39 (64.25–135.79)	102.18 ± 16.22 (73.50–148.00)	0.819
PA-baseline (mm)	42.52 ± 2.50 (37.60–47.30)	43.57 ± 2.73 (36.35–46.90)	41.05 ± 2.73 (38.60–49.75)	0.281
DPA-baseline (mm)	2.25 ± 0.96 (0.58–4.00)	1.49 ± 1.27 (0.01–3.83)	2.94 ± 1.06 (0.56–4.57)	0.020 ^(I–II)^ *
PA–DPA (mm)	38.37 ± 2.50 (32.90–42.60)	39.16 ± 2.54 (3.75–44.50)	36.23 ± 8.55 (3.75–44.55)	0.030 ^(I–II)^ *
α–β (mm)	13.96 ± 5.29 (5.79–25.30)	11.64 ± 6.03 (3.64–26.25)	14.21 ± 5.58 (3.64–26.25)	0.032 ^(II–III)^ *
Maxillary sinus width (mm)	14.21 ± 2.15 (10.20–22.47)	15.54 ± 3.18 (10.20–22.47)	15.10 ± 7.51 (7.62–47.04)	0.603

DPA: Descending palatine artery. PA: Piriform aperture. ∠ = angle between anatomical landmarks. * *p* < 0.05. Comparisons in parentheses (e.g., (II–I, III)) show where the significant difference lies. For example, (II–I, III) indicates that Class II patients differ significantly from both Class I and Class III groups. Statistical comparisons were performed using ANOVA. Post-hoc analyses were conducted with Bonferroni correction when variances were homogeneous and Tamhane’s T2 test when variances were unequal.

**Table 4:** Effect of skeletal patterns on variables.

Test Result Variable(s)	Cut Off	OR	Asymptotic 95% Confidence Interval		*p*-Value
			Lower Bound	Upper Bound	
Maxillary sinus volume	29.28	1.079	0.295	0.607	0.533
Maxillary sinus area	78.67	10.80	0.246	0.550	0.195
IV–VI	9.90	4.08	0.287	0.600	0.474
V–V′	3.44	2.74	0.048	0.836	0.015 *
VII–VII′	15.00	5.77	0.480	0.781	0.097
VIII–VIII′	12.90	2.79	1.043	1.958	0.000 *

OR: Odds ratio. * *p* < 0.05.

## Data Availability

The data that support the findings of this study are available on request from the corresponding author. The data are not publicly available due to privacy or ethical restrictions.

## References

[1] Kreeft A.M., Smeele L.E., Rasch C.R.N., Hauptmann M., Rietveld D.H.F., Leemans C.R., Balm A.J.M. (2012). Preoperative Imaging and Surgical Margins in Maxillectomy Patients. Head Neck.

[2] McMahon J.D., Wong L.S., Crowther J., Taylor W.M., McManners J., Devine J.C., Wales C., MacIver C. (2013). Patterns of Local Recurrence after Primary Resection of Cancers That Arise in the Sinonasal Region and the Maxillary Alveolus. Br. J. Oral Maxillofac. Surg..

[3] Standring S. (2021). Head and Neck: Overview and Surface Anatomy. Gray’s Anatomy: The Anatomical Basis of Clinical Practice.

[4] Parameswaran A., Juliet M., Thomas T.K., Ramanathan M., Mori Y. (2022). Evaluating Morphology of the Pterygomaxillary Junction and Its Association with the Orbit in Different Facial Skeletal Relationships. J. Oral Maxillofac. Surg..

[5] Cheung L.K., Fung S.C., Li T., Samman N. (1998). Posterior Maxillary Anatomy: Implications for Le Fort I Osteotomy. Int. J. Oral Maxillofac. Surg..

[6] Obata K., Kanemoto H., Umemori K., Ono K., Yoshioka N., Nishiyama A., Iwanaga J., Ibaragi S. (2024). Does the Anatomy around the Pterygomaxillary Suture Contribute to the Risk of Bad Fractures in Le Fort I Osteotomy?. J. Cranio-Maxillofac. Surg..

[7] Tunis T.S., Dratler S., Kats L., Allon D.M. (2023). Characterization of Pterygomaxillary Suture Morphology: A CBCT Study. Appl. Sci..

[8] Joshi R.J., AlOtaibi N., Naudi K., Henderson N., Benington P., Ayoub A. (2022). Pattern of Pterygomaxillary Disarticulation Associated with Le Fort I Maxillary Osteotomy. Br. J. Oral Maxillofac. Surg..

[9] Dadwal H., Shanmugasundaram S., Krishnakumar Raja V.B. (2015). Preoperative and Postoperative CT Scan Assessment of Pterygomaxillary Junction in Patients Undergoing Le Fort I Osteotomy: Comparison of Pterygomaxillary Dysjunction Technique and Trimble Technique—A Pilot Study. J. Maxillofac. Oral Surg..

[10] Lee S.H., Lee S.H., Mori Y., Minami K., Park H.S., Kwon T.G. (2011). Evaluation of Pterygomaxillary Anatomy Using Computed Tomography: Are There Any Structural Variations in Cleft Patients?. J. Oral Maxillofac. Surg..

[11] Odabaşı O., Erkmen E., Üçok C.Ö., Bakir M.A., Keriş E.Y., Şahin O. (2021). Morphometric Analysis of Pterygomaxillary Region by Using Cone Beam Computed Tomography. J. Stomatol. Oral Maxillofac. Surg..

[12] Ueki K., Hashiba Y., Marukawa K., Nakagawa K., Okabe K., Yamamoto E. (2009). Determining the Anatomy of the Descending Palatine Artery and Pterygoid Plates with Computed Tomography in Class III Patients. J. Cranio-Maxillofac. Surg..

[13] Ayhan M., Taşyapan S.A., Kundakçıoğlu A., Kasapoğlu M.B., İşler S.C., Aydil B.A., Özcan İ., Doğancalı G.E. (2023). Radiological Examination of the Relationship between the Pterygomaxillary Junction and Fracture Pattern. Ulus. Travma Acil Cerrahi Derg..

[14] Icen M., Orhan K., Oz U., Horasan S., Avsever H. (2020). Relationship between Pterygomaxillary Fissure Morphology and Maxillary/Mandibular Position: A Cone Beam Computed Tomography Assessment. J. Orofac. Orthop..

[15] Icen M., Orhan K. (2019). Cone-Beam Computed Tomography Evaluation of the Pterygomaxillary Fissure and Pterygopalatine Fossa Using 3D Rendering Programs. Surg. Radiol. Anat..

[16] Akın T., Eberliköse H., Tuğtağ Demir B., Bilecenoğlu B., Karasu H.A. Morphological Variations of the Pterygomaxillary Suture According to Skeletal Patterns. Proceedings of the XXIX International Symposium on Morphological Sciences (ISMS 2025).

